# Diabetes Mellitus and Pancreatic Ductal Adenocarcinoma—Prevalence, Clinicopathological Variables, and Clinical Outcomes

**DOI:** 10.3390/cancers14122840

**Published:** 2022-06-08

**Authors:** Anna Badowska-Kozakiewicz, Marta Fudalej, Daria Kwaśniewska, Marek Durlik, Anna Nasierowska-Guttmejer, Agata Mormul, Emilia Włoszek, Aleksandra Czerw, Tomasz Banaś, Andrzej Deptała

**Affiliations:** 1 Department of Cancer Prevention, Medical University of Warsaw, 01-445 Warsaw, Poland; abadowska@wum.edu.pl (A.B.-K.); marta.fudalej@wum.edu.pl (M.F.); 2 Department of Oncology and Haematology, Central Clinical Hospital of the Ministry of Interior and Administration, 02-507 Warsaw, Poland; daria.kwasniewska@cskmswia.gov.pl; 3 Department of Gastroenterological Surgery and Transplantation, Central Clinical Hospital of the Ministry of Interior and Administration, 02-507 Warsaw, Poland; marek.durlik@cskmswia.gov.pl; 4 Department of Pathology, Central Clinical Hospital of the Ministry of Interior and Administration, 02-507 Warsaw, Poland; anna.nasierowska@cskmswia.gov.pl; 5 Students’ Scientific Organization of Cancer Cell Biology, Department of Cancer Prevention, Medical University of Warsaw, 01-445 Warsaw, Poland; s074556@student.wum.edu.pl (A.M.); s081087@student.wum.edu.pl (E.W.); 6 Department of Health Economics and Medical Law, Medical University of Warsaw, 02-091 Warsaw, Poland; aleksandra.czerw@wum.edu.pl; 7 Department of Economic and System Analyses, National Institute of Public Health NIH-National Research Institute, 00-791 Warsaw, Poland; 8 Department of Gynecology and Oncology, Jagiellonian University Medical College, 31-501 Cracow, Poland; tomasz.1.banas@uj.edu.pl; 9 Department of Radiotherapy, Maria Sklodowska-Curie Institute–Oncology Centre, 31-115 Cracow, Poland

**Keywords:** pancreatic cancer, diabetes mellitus, oncology

## Abstract

**Simple Summary:**

The aim of this study is to describe the prevalence of diabetes mellitus (DM) among patients with the diagnosis of pancreatic ductal adenocarcinoma (PDAC), analyse the association between the occurrence of DM and clinicopathological factors, and detect variables influencing overall survival. Diabetes mellitus is prevalent among patients with pancreatic cancer. In our study, patients with diabetes mellitus receiving palliative chemotherapy had significantly higher median OS than those without. Among variables influencing survival, TNM stage, nodal involvement, tumour site, levels of CEA and CRP were confirmed.

**Abstract:**

Background: pancreatic ductal adenocarcinoma (PDAC) is the seventh leading cause of cancer-related deaths with increasing incidence and link to the onset of diabetes mellitus (DM). The aim of this study is to describe the prevalence of DM among patients with the diagnosis of PDAC, analyse the association between the occurrence of DM and clinicopathological factors, and detect variables influencing overall survival. Methods: a retrospective analysis of medical records was performed. The patients were divided into non-DM (*n* = 101) and DM (*n* = 74) groups. Statistical analysis with the usage of appropriate tests was conducted. Results: Patients in the groups of DM and NODM had significantly longer median OS than the non-DM group. Nodal involvement, tumour location, level of CEA, CRP and CRP/lymphocytes ratio were significantly associated with OS among patients with any type of DM. Neutropenia was less frequently observed in the DM group. Conclusions: DM is prevalent among patients with pancreatic cancer. In our study, patients with DM receiving palliative chemotherapy had significantly higher median OS than those without DM. The increased comprehension of the mechanisms of the relationship between DM and pancreatic cancer needs further research, which might provide avenues for the development of novel preventive and therapeutic strategies.

## 1. Introduction

Pancreatic cancer (PC) is the seventh leading cause of cancer-related deaths worldwide. Unlike other malignancies, incidence continues to increase, with a slight improvement in survival rates [1,2]. Specifically, pancreatic ductal adenocarcinoma (PDAC) is the most common pancreatic malignancy, representing over 90% of the pancreatic lesions [3]. Complete surgical resection provides the only chance for a cure; however, only 20% of patients are diagnosed with resectable disease. Additionally, 80% of surgically resected PDACs experience recurrence within five years of the resection [4]. PC patients’ overall 5-year survival rate is <5% [5]. Poor prognosis is associated with several factors, encompassing diagnosis at an advanced stage, early distant metastases, remarkable resistance to most conventional treatment options and a dense tumour microenvironment [3]. Identifying risk factors might lead to the earlier detection of pancreatic cancer and a more favourable prognosis. One potentially significant risk factor for this malignancy is diabetes mellitus (DM) [6]. Type 2 diabetes mellitus (T2DM) is the most prevalent form of diabetes, estimated for approximately 90% of diabetic patients. Hyperglyceamia results from resistance to insulin action combined with inadequate insulin secretion [7]. Clinical and experimental analysis revealed that pancreatic cancer is frequently linked to the onset of DM [8]. Studies confirm that the highest risk for PDAC is observed within the first two years after diabetes diagnosis [9]. Moreover, surgical procedures, both Whipple and distal resection, might lead to new-onset diabetes mellitus (NODM); however, the exact risk of this complication is unknown [10]. NODM is defined as a disease caused by the loss or destruction of the pancreatic endocrine parenchyma [7].

Despite the close relationship between DM and PDAC, little is known about the exact prevalence and impact of T2DM and NODM on clinical outcomes in PDAC [11]. Available information concerning this subject is limited and inconsistent. Various studies suggested that DM did not significantly affect overall survival (OS), whereas others found that DM significantly reduced survival [12].

Our study aims to describe the prevalence of diabetes mellitus among patients diagnosed with PDAC, analyse the association between the occurrence of DM and clinicopathological factors and detect variables influencing overall survival (OS).

## 2. Materials and Methods

### 2.1. Patients and Data Collection

**Figure 1:**
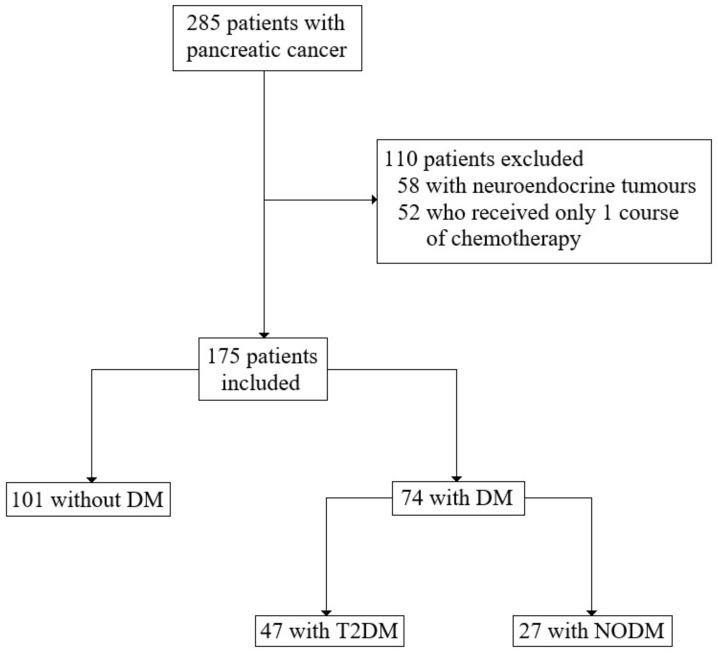
Summary of study design with inclusion and exclusion criteria.

We retrospectively analysed the medical history of 285 patients with the diagnosis of pancreatic cancer [C25 according to the International Statistical Classification of Diseases and Related Health Problems (ICD-10)], who were treated in the Department of Oncology and Haematology and Department of Gastroenterological Surgery and Transplantation at the Central Clinical Hospital (CSK) of the Ministry of Interior and Administration (MSWiA) in Warsaw, Poland between February 2012 and March 2021. After excluding 52 patients with neuroendocrine tumours and 58 patients who received only one course of chemotherapy, 175 patients were included in the study for the analysis (Figure 1). The analysed medical data encompassed sex, age, ECOG, other diseases (diabetes, hypertension, immunological and malignancies), pathological variables (tumour site, tumour size, histological grading, nodal involvement, tumour stage, neuro- and angioinvasion and resection margin), treatment data (type of the operation, vascular reconstruction, postoperative complications, adjuvant and palliative chemotherapy with side effects), laboratory findings, survival and progression time.

### 2.2. Study Design

The patients were divided into two following groups: patients without diabetes mellitus (non-DM) (*n* = 101) and patients with DM (*n* = 74). The DM group was further sub-divided into T2DM and NODM. Diabetes mellitus was diagnosed according to the below criteria:

(i) Two consecutive fasting glucose levels ≥140 mg/dL (7.8 mmol/L);
(ii) Random plasma glucose ≥200 mg/dL (11.1 mmol/L) in patients with classic symptoms of hyperglycaemia or hyperglycaemic crisis; or
(iii) 2-h plasma glucose ≥200 mg/dL (11.1 mmol/L) during an oral glucose tolerance test (OGTT).

Tumour staging was performed according to the American Joint Cancer Committee (AJCC) Staging Manual, 8th edition. Deaths were identified by reviewing the medical records. Recurrence was detected by abdominal and chest computed tomography (CT) during the follow-up period. The study focused on the DM group—the non-DM group was enrolled for the comparison concerning clinicopathological variables.

### 2.3. Statistical Analysis

Statistical analysis was conducted with the usage of IBM SPSS Statistics 27. All analysed variables are presented as mean and standard deviation or frequency with percentages. Differences in categorical variables were assessed as appropriate by either Chi-square or Fisher-exact test. The Student’s test and one-way analysis of variance were used to compare continuous variables. Survival (presented as median value) was calculated from the time of diagnosis of pancreatic cancer to the time of death from any cause. Alive patients were censored at their last follow-up. Survival was estimated using the Kaplan–Meier method and compared using the log-rank test. The Cox proportional hazards model was used to determine the prognostic factors in the univariate analysis of survival rates. The prognostic factors detected in univariate analyses as statistically significant were analysed further with multivariate Cox regression. The analysis was performed using the backward method based on the Wald statistic. In each step of this method, one prognostic factor with the weakest association with survival was excluded. The multivariate analyses allowed for indicating the strongest prognostic factor. A *p*-value of ≤0.05 (two-sided) was regarded as statistically significant in all analyses.

### 2.4. Ethics Approval and Consent to Participate

The work described in this article has been carried out in accordance with the Code of Ethics of the World Medical Association (Declaration of Helsinki) on medical research involving human subjects, which is the ethical principles defined in the Farmington Consensus of 1997. The study was acknowledged by the Bioethics Committee of the Medical University of Warsaw (AKBE/144/2022).

## 3. Results

Seventy-four patients with both DM and PDAC were enrolled in the analysis. Forty-seven of them were diagnosed with type 2 diabetes, while 27 developed NODM after the surgery (which accounted for 21.2% of all operated patients in the study). The majority of patients with DM were men (54.1%) with ECOG 1 status (71.6%). The mean age of the patients was 64.3 [standard deviation (SD) 8.2, range: 40–87]. Most of the patients were diagnosed with PDAC in the head of the pancreas (77.0%), in the IIB stage (29.7%), and with grading 2 (57.2%). Nodal involvement was confirmed in 33.9% of cases. Neuroinvasion was confirmed in 87.5% of the analysed samples, while angioinvasion in 69%.

Concerning other diseases, 9 patients were diagnosed with autoimmune disease (8—hypothyroidism, 1—rheumatoid arthritis), 45 with hypertension, and 8 with other cancers, encompassing prostate, breast, ovarian and colorectal malignancies. Forty-eight patients received adjuvant chemotherapy, and 74.5% of them developed adverse effects, among which neutropenia was the most common one. Thirty-one patients (64.6%) in the adjuvant group experienced disease progression and further received palliative therapy. Twenty-six patients received palliative chemotherapy from the beginning. In total, 57 patients eventually received palliative treatment, and 71.9% of them developed adverse effects, among which neutropenia was the most common one. Concerning T2DM treatment, 22 patients received metformin, 23 insulin, 1 empagliflozin, and 1 sulphonylurea.

Statistical analysis comparing the non-DM group with other studied groups is presented in Table 1. Hypertension was more often diagnosed among the DM group (*p* < 0.024) and T2DM group (*p* < 0.011) than in the non-DM group. In the laboratory findings, patients with DM had a lower CRP/lymphocytes (CLR) ratio before the first course of the chemotherapy (*p* < 0.050). Neutropenia as the side effect of adjuvant chemotherapy was more frequently observed in the non-DM group than in the DM group (*p* < 0.034) and NODM group (*p* < 0.013). On the other hand, neutropenia as the side effect of palliative chemotherapy was more frequently observed in the non-DM group than in the DM 2 group (*p* < 0.020). There were no significant differences in the pathological variables.

**Figure 2:**
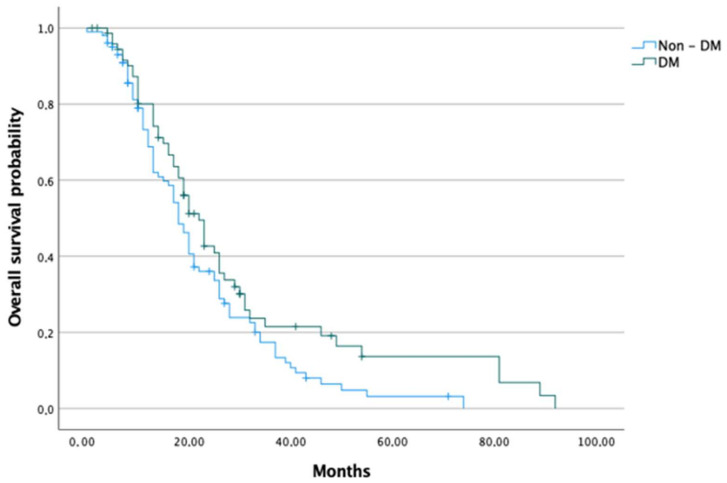
Overall survival of pancreatic cancer patients in DM and non-DM groups.

**Figure 3:**
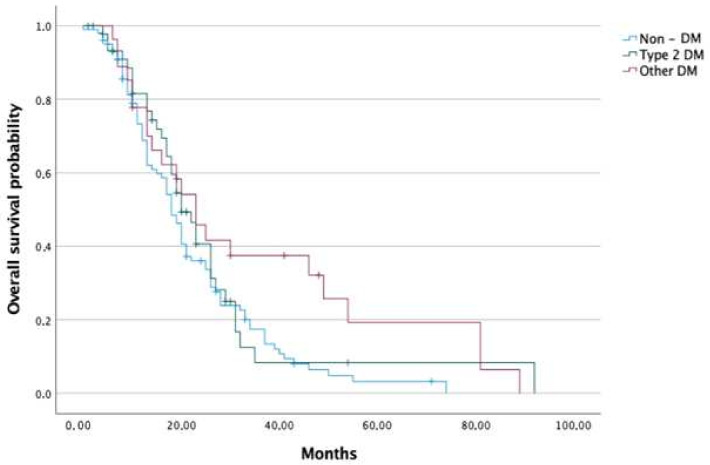
Overall survival of pancreatic cancer patients in the non-DM, T2DM and NODM groups.

Median OS was 18, 22 20, and 23 months in the non-DM, DM, T2DM and NODM groups, respectively. Patients in the groups of DM and NODM had significantly higher median OS than the non-DM group (*p* < 0.050 and 0.017, respectively). Analysed groups were further sub-divided into a group receiving adjuvant chemotherapy and a group receiving palliative chemotherapy (presented with advanced disease at the time of diagnosis). Regarding adjuvant chemotherapy, no difference in survival between non-DM and DM groups was detected; nevertheless, the NODM group had a significantly higher median OS than the non-DM group (26.5 months vs. 20.5 months, *p* < 0.028). In terms of palliative chemotherapy, patients with DM had significantly higher median OS than those without DM (18 months vs. 13 months, *p* < 0.034) (Figure 2 and Figure 3).

No significant differences concerning DFS and PFS were confirmed (Table 1).

In the univariate analysis for survival in all DM patients, nodal involvement (N2 stage) (*p* = 0.020), tumour location (*p* = 0.050), level of CEA (*p* = 0.019), CRP (*p* < 0.001) and CLR (*p* = 0.001) were significantly associated with OS (Table 2). These prognostic factors were analysed further in multivariate Cox regression using the backward method based on Wald statistics. The results are depicted in Table 3. Out of five prognostic factors analysed, the CRP level was the last excluded, meaning it was the strongest predictor of survival in the DM group.

In NODM group, only the TNM stage was independent prognostic factor for survival (*p* < 0.015, HR 1.774 95% CI 1.116–2.822) (Table 4). The multivariate analysis was not performed in this case because there was only one statistically significant prognostic factor.

In T2DM group, nodal involvement (N2 stage) (*p* = 0.023), CRP level (*p* < 0.001) and CLR (*p* = 0.004) were significantly associated with OS (Table 5). The results of subsequent multivariate analysis are depicted in Table 6. Out of three prognostic factors analysed, the CRP level again was the last one excluded, meaning it was the strongest predictor of survival in the T2DM group.

## 4. Discussion

Our study focused on various aspects to characterize patients with both DM and PC. We investigated not only oncological variables but also pathological and surgical ones. In the analysis, we incorporated data about chemotherapy schemes with side effects and also DM medicaments. Pancreatic cancer and DM are bidirectionally associated—DM is proved to be both the cause and consequence of PDAC [13]. In the analysed sample, 42.3% of patients developed DM—type 2 before the PDAC diagnosis or new-onset DM after the surgery. These results are in line with previous ones, in which the prevalence of DM among PDAC patients is estimated to reach 40–65% [11]. Except for PDAC, the prevalence of diabetes in other malignancies is similar to that of healthy controls [14]. Various mechanisms are responsible for the strong correlation between DM and PDAC. Clinical studies proved that the β cell response (measured by response to an oral glucose load, hyperglycaemic clamp or glucagon stimulation) is impaired in patients with PDAC [14]. Insulin released by β cells might indirectly promote carcinogenesis via acting on the growth hormone/insulin-like growth factor (GH/IGF) axis and increasing levels of free and bioactive insulin-like growth factor-1 (IGF-1) [15]. Both insulin and redundant IGF-1 reveal trophic effects on insulin receptors on acinar cells and IGF-I receptors present in any nearby cells, including transformed ones. As a result, insulin and IGF-1 promote their survival and proliferation, thus possibly contributing to the observed increased risk of developing PDAC [16]. Moreover, inadequate glycaemic control leads to producing increased levels of advanced glycation end products (AGE). AGE activate their receptors, which bind certain other ligands (inflammatory cytokines and S100 family) that are implicated in inflammation and PDAC progression [17].

Our study revealed no significant differences in the pathological variables between DM and non-DM groups. In the study by Hank et al. [18] (2019) conducted on patients undergoing pancreatic resection, diabetic patients had significantly larger tumours, increased lymph-node involvement and higher rates of perineural and lympho-vascular invasion. Chu et al. [19] (2010) proved tumour size as the only pathological variable differentiating DM and non-DM groups. Ben et al. [20] (2012) detected that the percentage of cases with neural invasion in those with DM was significantly higher than those without DM.

Oncological patients with pre-existing diabetes have 50% higher post-operative mortality rates, probably due to greater inflammation risk [19,21]. On the other hand, consistently with previous findings, in this study, DM was not associated with a higher rate of post-surgical complications [18,22,23]. However, Chu et al. [22] (2010) suggested that the role of PDAC-associated DM as a risk factor for postresection pancreatic fistula should be further explored, as in their study DM patients had a significantly higher likelihood of developing fistulas. Pancreatic cancer related-DM improves after the resection of tumour mass despite surgical removal of a variable amount of pancreatic tissue, which supports the hypothesis that pancreatic carcinoma cells induce DM themselves [6].

Reports concerning the exact impact of DM on the overall survival of patients with PC are ambiguous. Our study revealed that patients with DM had significantly higher median OS than those without DM. After subdividing patients into those receiving adjuvant or palliative chemotherapy, DM positively impacted survival only among patients with advanced disease. However, DM treatment with neither insulin nor metformin served as a prognostic factor for OS. Numerous studies suggested that DM is associated with reduced OS [6,18,20,24,25,26]. On the contrary, other studies proved that DM does not affect pancreatic cancer OS or even improve survival [27,28,29,30,31]. The meta-analysis conducted by Mao et al. [12] (2015) suggested the negative effect of DM on survival primarily among patients with resectable tumours but not in those with late-stage disease. Chu et al. [19] proved that compared with non-DM, NODM was independently associated with survival reduction after PDAC resection; nevertheless, a reduction in patients with longstanding DM versus those without DM turned out to be no longer statistically significant. Choi et al. [32] (2016) revealed that individuals with advanced PC and DM tend to survive longer than those without DM. They linked this phenomenon to metformin usage, which conferred a prognostic benefit. Moreover, in the paper introduced during the 2013 ASCO Annual Meeting I, it was suggested that in patients with advanced tumours, recent-onset DM and metformin treatment are positive prognostic indicators associated with longer OS [33]. Various preclinical studies demonstrated a positive impact of metformin on PC through increasing the chemosensitivity of PC cells to gemcitabine [34], presenting an anti-tumour effect in combination with liraglutide [35] synergistically with pitavastatin activating apoptosis and autophagy in PC cells [36], or finally inhibiting tumour growth, and prolonging the overall survival in mice model [37]. Concerning insulin, the recent study by Pretta et al. [38] (2021) revealed that insulin-treated patients compared with non-DM had a significantly increased survival in the multivariate analysis. The role of insulin in carcinogenesis is disputed. According to preclinical and clinical trials, it is generally considered to promote tumour growth; however, there is no unanimity, as Pircher et al. [39] (2018) proved that insulin might suppress the activation of mTOR and inhibit tumour growth.

Several studies reported that tumour sites influenced survival. Our study aligns with these findings and confirms that patients with PDAC located in the head have a worse prognosis. The various analysis also suggested that pancreatic body and tail locations were independent indicators for better survival [40,41]. It might be linked to the pancreatic head’s more complex lymphatic drainage system and is more frequently associated with higher nodal involvement [42,43]. Moreover, it could be correlated with intervention differences. The resection of distal pancreatic tumours is safer and more feasible [44]. Body or tail location does not lead to malignant biliary obstruction and thus is not associated with potential biliary drainage and further complications delaying appropriate surgery [45]. Contrastingly, a few studies suggested better outcomes among patients with head tumours. The authors explained it with an additional period resulting from an earlier diagnosis rather than tumour biology or differences in interventions [41].

Neuropathy is a common adverse effect associated with gemcitabine-nab-paclitaxel and mFOLFIRINOX chemotherapy schemes [46,47]. On the other hand, DM is the most common cause of autonomic neuropathy; nevertheless, the pathophysiology of diabetic neuropathy is not fully understood, and its aetiology seems to be multifactorial [48]. Interestingly, our study revealed that patients with DM are not more likely to develop neuropathy as a side effect of palliative chemotherapy. To our best knowledge, this is the first analysis comparing the prevalence of neuropathy as a chemotherapy’s side effect between patients with and without DM. Some studies confirmed only a significantly higher prevalence of perineural invasion among patients with DM [19,49]. Jian et al. [50] (2020) analysed genes associated with PDAC and involved in diabetic neuropathy progression. They selected matrix metalloproteinase-9 (MMP9) as a prognostic marker for pancreatic cancer and possible adjustment to the treatment of diabetes, pancreatic cancer and associated neuropathy.

The risk of PDAC declines with the increasing duration of DM [15]. This phenomenon might be linked to possible lifestyle changes and selected glucose-lowering medication usage. Various epidemiologic studies confirmed the association of metformin use with a reduced incidence of PDAC among patients with DM; however, the published reports seem to not be universally consistent [51]. Its anticancer effect might be associated with direct actions on transformed pancreatic cells and systemic actions. Metformin inhibits synthesis of mitochondrial adenosine triphosphate (ATP), leading to insufficient energy production. As a result, it increases cellular adenosine monophosphate/adenosine triphosphate (AMP/ATP) and adenosine diphosphate/adenosine triphosphate (ADP/ATP) ratios and activates AMP-activated protein kinase (AMPK). Stimulated AMPK inhibits the synthesis of macromolecules essential for further cell growth and inhibits the mechanistic target-of-rapamycin complex (mTORc), responsible for activating various cellular pathways [52,53]. Lower blood glucose leads to decreased mitogenic insulin secretion and further attenuates cell division [54]. In the studies conducted on genetic mice models, metformin inhibited cancer initiation, suppressed chronic pancreatitis-induced tumorigenesis and presented promising therapeutic effect in PDAC [37,55,56].

Various studies confirm that cancer progression is stimulated by systemic and local inflammatory reactions [57]. The immune system might suppress tumour development or progression by destroying cells with mutations; however, it can also promote pancreatic cancer progression by establishing favourable conditions for immunosuppression and further metastasis [58]. In terms of pancreatic cancer, one of the widely described inflammation-based parameters is CRP/albumin ratio (CAR). CAR was established as a strong prognostic and predictive factor in resectable and advanced tumours [59,60,61]. Lately, Fan et al. [62] (2020) have suggested elevated CLR as an independent risk factor for poor OS among PDAC patients. Another study by Strijker et al. [63] (2021) detected that CRP combined with CA19-9, albumin and LDH had a prognostic value, which was at least similar to that of ECOG performance status. The level of CLR is suspected to reflect the state of equilibrium between the systemic inflammatory and immunologic response. In our study, both higher CLR and higher CRP levels alone were significantly associated with worse OS among all patients with DM. Our multivariate analysis suggested CRP level as the strongest predictor of survival. CRP was previously claimed to associate with survival and cachexia among patients with PDAC [64,65]. Although some analyses also suggest that a reduced number of circulating white blood cells might present prognostic implications in PDAC patients, in our study, leukopenia alone revealed no correlation with survival [66].

Our analysis revealed that among patients with PDAC, those with DM are significantly more likely to develop hypertension, which is consistent with known risk factors for type II DM. Epidemiological studies have proven that metabolic syndrome and its components (hypertension, insulin resistance, central obesity, decreased levels of high-density lipoproteins (HDL) cholesterol and elevated triglyceride levels) may independently or in combination increase the risk of many types of cancer, including PDAC [67,68]. Moreover, some studies suggest that obesity significantly reduces OS among PDAC patients [23]. Our study did not include BMI in the analysis to compare. Xia et al. [69] (2020) suggested a potential joint effect of CRP and metabolic syndrome in pancreas tumorigenesis. The disruption of the inflammatory system is involved in the development of pancreatic cancer, and metabolic syndrome is related to pancreatic cancer risk, with diabetes being the critical component [70]. Elevated CRP was proven to be positively associated with the presence of the metabolic syndrome.

This study meets with some limitations. Firstly, it was a single-centre study. The juxtaposition of results obtained in the different clinical centres would present a broader view of the discussed subject. Secondly, it is a retrospective study and thus may present some potential selection bias. The duration of DM was not directly reported due to the encountered lack in the past medical history. Nonetheless, we firmly believe that outcomes acquired in our centre are a meaningful puzzle piece in the knowledge concerning the relationship between diabetes mellitus and pancreatic cancer.

## 5. Conclusions

Diabetes mellitus is prevalent among patients with pancreatic cancer. The results concerning DM impact on PDAC patients’ survival are contradictory; however, in our study, patients with DM receiving palliative chemotherapy had significantly higher median OS than those without DM. Regarding adjuvant chemotherapy, no difference in survival between non-DM and DM groups was detected; nevertheless, we have observed that the NODM patients had a significantly longer median OS than the non-DM patients. Among variables influencing survival, TNM stage, nodal involvement, tumour site, levels of CEA and CRP were confirmed, with CRP level being the strongest one. As an adverse effect of chemotherapy, neutropenia affects patients with DM less often. The increased comprehension of the mechanisms of the relationship between DM and pancreatic cancer needs further research, which might provide avenues for developing novel preventive and therapeutic strategies.

## Figures and Tables

**Table 1:** Characteristics of the three study groups and comparison to the non-DM group.

Variable	Non-DM (*n* = 101)	DM (*n* = 74)		T2DM (*n* = 47)		NODM (*n* = 27)	
	M ± SD/*n* (%)/ MD (95% CI)	M ± SD/*n* (%)/ MD (95% CI)	p *	M ± SD/*n* (%)/ MD (95% CI)	p **	M ± SD/*n* (%)/ MD (95% CI)	p ***
Gender (male)	47 (46.5%)	40 (54.1%)	0.326	27 (57.4%)	0.216	13 (48.1%)	0.881
Age (years)	63.41 ± 10.68	64.30 ± 8.23	0.550	65.66 ± 7.99	0.200	61.93 ± 8.26	0.506
ECOG (0/1/2)	6/75/17	6/54/14	0.927	5/35/7	0.893	1/20/6	0.441
History of other cancers	9 (8.9%)	8 (11.1%)	0.632	7 (15.2%)	0.255	1 (3.8%)	0.686
Autoimmune disease	11 (10.9%)	9	0.936	7	0.717	2	1.000
Hypertension	44 (43.6%)	45 (60.8%)	**0.024**	31 (66.0%)	**0.011**	14 (51.9%)	0.442
Adverse effects—adjuvant chemotherapy	44 (83.0%)	35 (74.5%)	0.295	24 (82.8%)	1.000	11 (61.1%)	0.099
Neurological	4 (7.5%)	2 (4.3%)	0.681	2 (6.9%)	1.000	0	0.566
Neutropenia	38 (71.7%)	24 (51.1%)	**0.034**	17 (58.6%)	0.228	7 (38.9%)	**0.013**
Hepatological	2 (3.8%)	4 (8.5%)	0.416	2 (6.9%)	0.612	2 (11.1%)	0.265
Adverse effects—palliative chemotherapy	67 (83.8%)	41 (71.9%)	0.095	24 (68.6%)	0.065	17 (77.3%)	0.531
Neurological	14(17.5%)	7 (12.3%)	0.403	4 (11.4%)	0.410	3 (13.6%)	1.000
Neutropenia	44 (55.0%)	22 (38.6%)	0.058	11 (31.4%)	**0.020**	11 (50%)	0.677
Hepatological	5 (6.3%)	6 (10.5%)	0.525	5 (14.3%)	0.170	1 (4.5%)	1.000
Operative complications	5 (7.0%)	3 (5.6%)	1.000	2 (6.3%)	1.000	1 (4.5%)	1.000
Neuroinvasion	43 (79.6%)	35 (87.5%)	0.315	24 (88.9%)	0.365	11 (84.6%)	1.000
Angioinvasion	46 (85.2%)	29 (69.0%)	0.058	21 (72.4%)	0.160	8 (61.5%)	0.110
Grading (1/2/3)	11/51/14	9/39/12	0.964	5/27/7	0.965	4/12/5	0.632
T (1/2/3/4)	2/15/51/5	1/15/34/2	0.689	1/11/19/2	0.519	0/4/15/0	0.825
N (0/1/2)	13/43/16	17/25/11	0.181	9/17/6	0.508	8/8/5	0.121
M (0/1)	56/45	52/22	0.046	32/15	0.145	20/7	0.080
TNM Stage (IA/IB/IIA/IIB/III/IV)	1/3/5/31/12/45	1/6/6/22/10/21	0.268	1/5/2/15/5/15	0.354	0/1/4/7/5/6	0.156
Localisation	77/9/7/3/2/3	57/4/5/0/4/4	0.475	33/3/5/0/3/3	0.414	24/1/0/0/1/1	0.579
Metastases	29/4/30/3/9	25/6/11/3/3	0.131	16/4/7/2/2	0.219	9/2/4/1/1	0.440
CEA > 5 ng/mL	22 (32.8%)	19 (36.5%)	0.673	15 (40.5%)	0.432	4 (26.7%)	0.765
CA19-9 > 37 IU/mL	51 (56.7%)	38 (58.6%)	0.824	26 (57.8%)	0.902	12 (60.0%)	0.785
CRP/lymphocytes ratio > 1.8	37 (66.1%)	21 (46.7%)	**0.050**	17 (50.0%)	0.131	4 (36.4%)	0.092
Lymphocytes > 1 × 10^3^/µL	93 (92.1%)	66 (91.7%)	0.922	42 (89.4%)	0.552	24 (96.0%)	0.687
Haemoglobin > 12 g/dL	63 (63.6%)	51 (70.8%)	0.324	33 (70.2%)	0.434	18 (72.0%)	0.432
Platelets > 400 × 10^3^/µL	18 (17.8%)	11 (15.3%)	0.659	6 (12.8%)	0.437	5 (20.0%)	0.777
CRP > 5 mg/L	28 (59.0%)	18 (40.0%)	0.316	15 (44.1%)	0.588	3 (27.3%)	0.167
OS	18 (15.7–20.3)	22 (18.4–26.6)	**0.050**	20 (15.9–24.1)	0.462	23 (16.0–30.0)	**0.017**
DFS	12 (9.0–15.0)	9 (7.7–10.3)	0.533	9 (4.9–13.1)	0.706	7 (4.9–9.1)	0.580
PFS	5 (3.9–6.1)	6 (4.34–7.6)	0.206	6 (4.6–7.4)	0.389	7 (4.7–9.3)	0.230

Abbreviations: Non-DM—group without diabetes mellitus, DM—diabetes mellitus, T2DM—type 2 diabetes mellitus, NODM—new-onset diabetes mellitus, M—mean, SD—standard deviation, *n*—number, MD—median, 95% CI—95% Confidence Interval, OS—overall survival, DFS—disease-free survival and PFS—progression-free survival. *p* * non-DM vs. DM, *p* ** non-DM vs. T2DM and *p* *** non-DM vs. NODM. Bolded value – value statistically significant.

**Table 2:** Univariate analyses of prognostic factors for overall survival—DM group.

Variable	HR (95% CI)	*p*-Value
Age (years)	0.986 (0.952–1.021)	0.424
Gender	-	-
Female	Ref	-
Male	1.194 (0.690–2.063)	0.526
Autoimmune disease	0.660 (0.260–1.676)	0.382
Hypertension	0.643 (0.372–1.112)	0.114
T	-	-
T1	Ref	-
T2	0.336 (0.041–2.729)	0.308
T3	0.254 (0.032–1.996)	0.192
T4	0.456 (0.040–5.222)	0.528
N	-	-
N0	Ref	-
N1	1.692 (0.768–3.730)	0.192
N2	3.163 (1.200–8.338)	**0.020**
M	-	-
M0	Ref	-
M1	1.524 (0.852–2.725)	0.156
TNM stage	-	-
IA	Ref	-
IB	0.310 (0.033–2.862)	0.301
IIA	0.149 (0.015–1.465)	0.103
IIB	0.302 (0.039–2.359)	0.254
III	0.551 (0.067–4.513)	0.579
IV	0.450 (0.058–3.450)	0.449
Angioinvasion	1.193 (0.525–2.712)	0.674
Neuroinvasion	0.563 (0.192–1.653)	0.296
R	-	0.081
R0	Ref	
R1	1.789 (0.930–3.442)	
Grading	-	-
G1	Ref	-
G2	1.691 (0.659–4.339)	0.275
G3	2.155 (0.711–6.531)	0.175
Tumour site	-	-
Head	Ref	-
Other	1.809 (0.999–3.277)	**0.050**
Operation type	-	-
Whipple	Ref	-
Other	1.367 (0.688–2.717)	0.372
Vascular reconstruction	1.242 (0.543–2.842)	0.608
Adverse effects—adjuvant chemotherapy	1.521 (0.650–3.557)	0.333
Neutropenia	1.673 (0.792–3.535)	0.178
Hepatological	1.113 (0.263–4.722)	0.883
Adverse effects—palliative chemotherapy	1.025 (0.544–1.931)	0.939
Neutropenia	0.651 (0.356–1.191)	0.163
Hepatological	1.182 (0.418–3.345)	0.751
Neurological	0.490 (0.173–1.386)	0.179
History of other cancers	1.907 (0.838–4.337)	0.124
CEA	1.004 (1.001–1.007)	**0.019**
CA 19-9	1.000 (0.999–1.002)	0.059
Lymphocytes	0.956 (0.844–1.082)	0.475
CRP/lymphocytes ratio	1.032 (1.013–1.053)	**0.001**
Haemoglobin	0.954 (0.720–1.263)	0.741
Platelets	1.001 (0.999–1.003)	0.249
Calcium	0.446 (0.051–3.899)	0.465
CRP	1.017 (1.008–1.025)	**<0.001**

Abbreviations: Ref–reference. Bolded value–value statistically significant.

**Table 3:** Multivariate analysis of prognostic factors for overall survival—DM group.

Variable	HR (95% CI)	*p*-Value
Step 1		
N	-	-
N0	Ref	-
N1	1.180 (0.182–7.667)	0.862
N2	1.239 (0.126–12.214)	0.854
Tumour site	-	-
Head	Ref	-
Other	1.149 (0.226–5.833)	0.867
CEA	1.006 (0.983–1.030)	0.620
CRP/lymphocytes ratio	0.984 (0.900–1.075)	0.718
CRP	1.027 (0.965–1.092)	0.401
Step 2		
Tumour site	-	-
Head	Ref	-
Other	1.030 (0.352–3.011)	0.957
CEA	1.006 (0.984–1.028)	0.621
CRP/lymphocytes ratio	0.988 (0.917–1.065)	0.756
CRP	1.024 (0.971–1.079)	0.381
Step 3		
CEA	1.005 (0.984–1.028)	0.623
CRP/lymphocytes ratio	0.988 (0.917–1.065)	0.988
CRP	1.024 (0.972–1.079)	0.369
Step 4		
CEA	1.005 (0.984–1.027)	0.633
CRP	1.016 (0.997–1.035)	0.095
Step 5		
CRP	1.017 (1.008–1.025)	**<0.001**

Abbreviations: Ref–reference. Bolded value–value statistically significant.

**Table 4:** Univariate analyses of prognostic factors for overall survival—NODM group.

Variable	HR (95% CI)	*p*-Value
Age	0.563 (0.229–1.383)	0.210
Gender	-	0.187
Female	Ref	
Male	0.517 (0.194–1.377)	
Hypertension	0.418 (0.164–1.069)	0.069
Nodal involvement	3.142 (0.808–12.216)	0.098
TNM stage (IA/IB/IIA/IIB/III/IV)	1.774 (1.116–2.822)	**0.015**
R	-	0.081
R0	Ref	
R1	1.287 (0.443–3.737)	

Abbreviations: Ref–reference. Bolded value–value statistically significant.

**Table 5:** Univariate analyses of prognostic factors for overall survival—T2DM group.

Variable	HR (95% CI)	*p*-Value
Age (years)	0.978 (0.926–1.033)	0.426
Gender	-	-
Female	Ref	-
Male	0.898 (0.639–1.263)	0.536
Autoimmune disease	0.962 (0.334–2.771)	0.942
Hypertension	0.732 (0.358–1.500)	0.393
T	-	-
T1	Ref	-
T2	0.268 (0.030–2.383)	0.238
T3	0.196 (0.025–1.702)	0.139
T4	0.358 (0.030–4.325)	0.419
N	-	-
N0	Ref	-
N1	1.383 (0.465–4.111)	0.560
N2	5.342 (1.264–22.564)	**0.023**
M	-	-
M0	Ref	-
M1	1.524 (0.852–2.725)	0.156
TNM stage	-	-
IA	Ref	-
IB	0.179 (0.017–1.900)	0.154
IIB	0.215 (0.025–1.827)	0.159
III	0.734 (0.079–6.829)	0.786
IV	0.255 (0.029–2.172)	0.211
Angioinvasion	2.023 (0.670–6.109)	0.212
Neuroinvasion	0.865 (0.194–3.863)	0.849
R	-	0.064
R0	Ref	
R1	2.185 (0.956–4.996)	
Grading	-	-
G1	Ref	-
G2	2.111 (0.494–9.014)	0.313
G3	1.433 (0.259–7.920)	0.680
Tumour site	-	-
Head	Ref	-
Other	1.705 (0.841–3.454)	0.139
Operation type	-	-
Whipple	Ref	-
Other	1.307 (0.567–3.184)	0.555
Vascular reconstruction	0.877 (0.295–2.606)	0.814
DM treatment	-	-
Insulin	Ref	-
Metformin	0.726 (0.363–1.451)	0.364
Adverse effects—adjuvant chemotherapy	0.833 (0.237–2.934)	0.776
Neutropenia	1.055 (0.376–2.957)	0.919
Adverse effects—palliative chemotherapy	0.745 (0.336–1.650)	0.468
Neutropenia	0.541 (0.215–1.259)	0.191
Hepatological	0.763 (0.228–2.556)	0.662
Neurological	0.224 (0.030–1.669)	0.144
History of other cancers	1.748 (0.695–4.396)	0.235
CEA	1.012 (0.978–1.048)	0.493
Lymphocytes	0.964 (0.840–1.107)	0.607
CRP/lymphocytes ratio	1.013 (1.004–1.022)	**0.004**
Haemoglobin	0.900 (0.639–1.263)	0.536
Calcium	0.738 (0.050–10.854)	0.825
CRP	1.018 (1.007–1.028)	**<0.001**

Abbreviations: Ref–reference. Bolded value–value statistically significant.

**Table 6:** Multivariate analyses of prognostic factors for overall survival—T2DM group.

Variable	HR (95% CI)	*p*-Value
Step 1		
N	-	-
N0	Ref	-
N1	0.620 (0.169–2.280	0.472
N2	2.116 (0.390–11.471)	0.385
CRP/lymphocytes ratio	0.998 (0.904–1.102)	0.971
CRP	1.017 (0.950–1.089)	0.629
Step 2		
N	-	-
N0	Ref	-
N1	0.616 (0.177–2.139)	0.445
N2	2.097 (0.416–10.562)	0.370
CRP	1.016 (0.995–1.037)	0.134
Step 3		
CRP	1.018 (1.007–1.028)	**<0.001**

Abbreviations: Ref–reference. Bolded value–value statistically significant.

## Data Availability

The data presented in this study are available on request from the corresponding author.

## Abbreviations

PDAC	Pancreatic ductal adenocarcinoma
PC	Pancreatic cancer
DM	Diabetes mellitus
NODM	New-onset diabetes mellitus
T2DM	Type 2 DM
OS	Overall survival
ICD-10	The International Statistical Classification of Diseases and Related Health Problems
CSK	Central Clinical Hospital
MSWiA	Ministry of Interior and Administration
ECOG	Eastern Cooperative Oncology Group performance status
non-DM	Group without diabetes mellitus
OGTT	Oral glucose tolerance test
AJCC	American Joint Cancer Committee
CT	Computed tomography
SD	Standard deviation
HR	Hazard ratio
CRP	C reactive protein
CLR	CRP/lymphocytes ratio
M	Mean
*n*	Number
MD	Median
95% CI	95% Confidence Interval
DFS	Disease-free survival
PFS	Progression-free survival
CEA	Carcino-embryonic antigen
CA19-9	Carbohydrate antigen 19-9
TNM	TNM Classification of Malignant Tumors
T	Tumour size
N	Lymph nodal involvement
M	Distant metastasis
R	Resection margin
GH/IGF	Growth hormone/insulin-like growth factor
IGF-1	Insulin-like growth factor-1
AGE	Advanced glycation end products
ASCO	American Society of Clinical Oncology
mTOR	The mammalian target of rapamycin
mFOLFIRINOX	A chemotherapy regimen: FOL—folinic acid
F	Fluorouracil (5-FU)
IRIN	Irinotecan
OX	Oxaliplatin
MMP9	Matrix metalloproteinase-9
AMP/ATP	Adenosine monophosphate/adenosine triphosphate
ADP/ATP	Adenosine diphosphate/adenosine triphosphate
AMPK	AMP-activated protein kinase
CAR	CRP/albumin ratio
HDL	High-density lipoproteins
